# New Onset Granulomatosis with Polyangiitis Associated with COVID-19

**DOI:** 10.1155/2021/8877292

**Published:** 2021-01-13

**Authors:** Moshe Y. Bressler, Naeha Pathak, Kelly Cervellione, Farshad Bagheri, Edward Epstein, Adnan Mir, Rebecca Tamez

**Affiliations:** ^1^New York Institute of Technology College of Osteopathic Medicine, Old Westbury, NY, USA; ^2^Jamaica Hospital, Department of Internal Medicine, Dermatology, New York, NY, USA; ^3^Jamaica Hospital, Department of Clinical Research, New York, NY, USA; ^4^Jamaica Hospital, Department of Internal Medicine, Infectious Disease, New York, NY, USA; ^5^Jamaica Hospital, Department of Internal Medicine, Nephrology, New York, NY, USA; ^6^Dermpath Diagnostics, New York Medical College Dermatology, Weill Cornell Medicine Dermatology, New York, NY, USA

## Abstract

The coronavirus disease 2019 (COVID-19) has recently been found to cause cutaneous vasculitis in patients. Granulomatosis with polyangiitis (GPA) is a type of small and medium vessel vasculitis that is often associated with pulmonary issues and has been shown to raise diagnostic complications in COVID-19 infection. In this report, we discuss the first case of new-onset GPA in the setting of active COVID-19 infection. Symptoms often overlap between the two diseases, and while there is no current cure for COVID-19, rapid immunosuppressive initiation can be lifesaving for patients with GPA. Thus, this case is essential in expanding our current knowledge of COVID-19 and its many skin manifestations.

## 1. Introduction

The coronavirus disease 2019 (COVID-19) pandemic caused by severe acute respiratory syndrome coronavirus 2 (SARS-CoV-2) has rapidly spread across the globe, while clinicians have been compelled to continuously expand their knowledge base of this novel complex disease and its associated symptoms. Several coronavirus patients have been found to have vascular endothelial damage, which is thought to play a role in the development of both skin lesions [1] and strokes [2]. Several dermatologists report seeing a vasculitis-like eruption in COVID patients [3, 4]. Herein, we discuss a case of new onset granulomatosis with polyangiitis (GPA), a small and medium vessel vasculitis, associated with a suspected case of COVID-19.

## 2. Case Presentation

**Figure 1:**
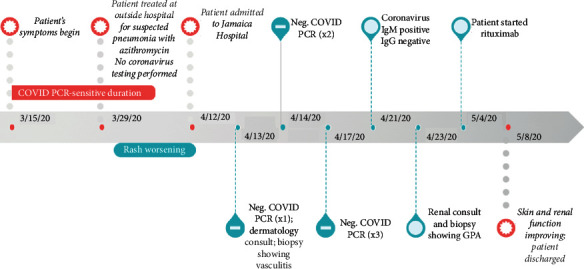
Timeline depicting onset of patient symptoms, test results, and disease course.

In April 2020, a 46-year-old man with a past medical history of diabetes presented to the emergency room with symptoms of cough, shortness of breath, and fevers for four weeks. He was treated for suspected pneumonia with azithromycin two weeks prior to presentation and denied any improvement in respiratory symptoms thereafter. In addition, he developed a painful and pruritic rash that was progressively worsening over the last two weeks (see Figure 1 for timeline of patient course).

**Figure 2:**
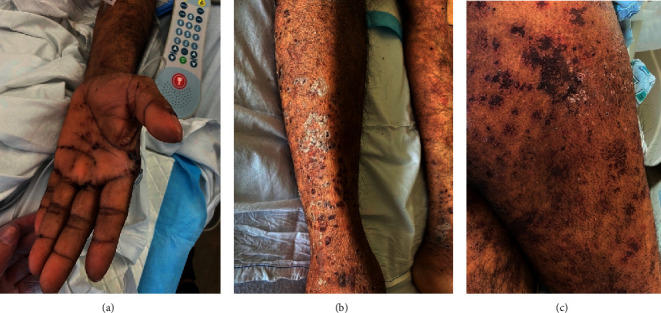
Cutaneous findings in a 46-year-old man with granulomatosis with polyangiitis and an active COVID-19 infection. (a) Purpuric macules and papules on palm and distal arm (b) Purpuric macules and papules with collarettes of scale on lower leg. (c) Retiform purpuric patches on thighs.

On physical examination, he was afebrile (36.9 C) with tachycardia (110 bpm), blood pressure 127/77 mm Hg, respiratory rate 20, and O_2_ saturation 94% on room air. He had erosions on bilateral nasal mucosa and vermillion lips with overlying crusts. Oropharynx was clear. There were purpuric macules and papules on bilateral palms, arms, and legs, few with overlying vesicular changes and collarettes of scale (Figures 2(a) and 2(b)). He also had retiform purpuric macules and patches on buttocks and thighs (Figure 2(c)), as well as ulcerations on buttocks at the gluteal cleft.

Laboratory evaluation showed an elevated white blood cell count (15.3K/*μ*L), decreased hemoglobin (10.3 g/dL), elevated blood urea nitrogen (48 mg/dL), creatine (2.9 mg/dL), and D-dimer (8,922 ng/mL). He was cANCA positive, coronavirus PCR negative via nasopharyngeal swab, and coronavirus antibody IgM positive and IgG negative. CT scan of the chest showed patchy ground-glass opacities in the lungs.

**Figure 3:**
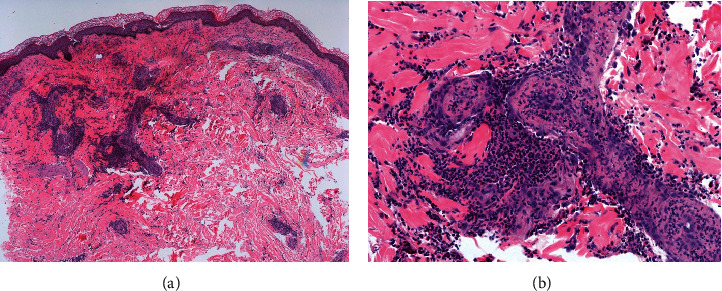
Histopathologic findings of the cutaneous lesions depicting leukocytoclasic vasculitis consistent with granulomatosis with polyangiitis. (a) Superficial and deep dermal perivascular inflammation with thrombus formation (H&E stain, 10x). (b) Extravasated erythrocytes and leukocytoclasia seen in the dermis with fibrinoid necrosis of vessels (H&E stain, 40x) and perivascular infiltrate composed of lymphocytes, histiocytes, neutrophils, and eosinophils.

Biopsy of skin showed superficial and deep perivascular inflammation with thrombus formation (H&E stain, 10x) (Figure 3(a)). High-power view showed an infiltrate composed of lymphocytes, histiocytes, neutrophils, and eosinophils, with prominent erythrocyte extravasation, leukocytoclasia, and fibrinoid necrosis of vessels (H&E stain, 40x) (Figure 3(b)). Renal biopsy showed interstitial mixed cell infiltrates and intraglomerular thrombi and necrosis, concerning for GPA.

Based upon his clinical presentation, histologic findings, and laboratory studies, the patient was diagnosed with GPA in the setting of suspected coronavirus infection. He was first treated for coronavirus with five-day course of hydroxychloroquine, followed by treatment of GPA with systemic steroids and rituximab infusion. Kidney function improved, and his rash completely resolved. He was discharged on a steroid taper.

## 3. Discussion

We discuss a case of c-ANCA-positive granulomatosis with polyangiitis possibly triggered by a COVID-19 infection. Our patient presented with four weeks of symptoms and had negative nasopharyngeal PCR test; however, positivity to RT-PCR begins to decline three weeks after initial symptom onset [5]. Furthermore, a review of 8,136 pooled specimens showed RT-PCR positivity using nasopharyngeal swabs to be a mere 45.5% (compared to bronchoalveolar lavage with 91.8%) [6]. Our patient showed positive IgM antibodies to COVID-19, owing to possible recent infection, although there has been a report of cross-reactivity to COVID-19 IgM serological testing in GPA [7]. We are hesitant to conclude this case as a false-positive, rather we hypothesize patient's delayed presentation and low-yield specimen for PCR testing may have missed an active or recent COVID-19 infection (as illustrated in Figure 1).

Infections or viruses can trigger GPA [8], and without treatment, it has a devastatingly high mortality rate [9]. First-line therapies are either rituximab or cyclophosphamide. Glucocorticoids can also be given, especially for maintenance therapy. In the setting of coronavirus, there is a concern that immunosuppressive therapies could increase the likelihood of adverse events including hospitalization and mortality [10, 11]. However, other studies suggest that the increased risk of COVID-19-related adverse events (such as admission to an intensive care unit or requiring mechanical ventilation) is minimal, and outcomes are improved when patients are on immune-modifying medications [10, 12]. Furthermore, patients given dexamethasone, a well-known immunomodulatory therapy, had significantly lower mortality and length of hospital stay [11].

## 4. Conclusion

GPA can mimic the clinical presentation of COVID-19 infection, and cross-reactivity can complicate interpretation of COVID-19 serology results. Prompt treatment for GPA is indicated to prevent irreversible lung and renal damage. While some immunotherapies may increase the risk of a COVID-19 infection, immunosuppressives have overall shown decreased risk of adverse events associated with infection and better patient outcomes. Our patient with biopsy-proven GPA in the setting of coronavirus infection improved clinically with the use of immunosuppressive therapy. We hope that, by presenting his case, we can further expand clinical knowledge about this complicated viral disease and the use of immunotherapy in a setting where the risk of coronavirus infection is high.
